# Multiple transcription factors involved in the response of Chinese cabbage against *Plasmodiophora brassicae*

**DOI:** 10.3389/fpls.2024.1391173

**Published:** 2024-06-06

**Authors:** Sida Meng, Xinyu Yan, Yinglan Piao, Shizhen Li, Xin Wang, Jing Jiang, Yue Liang, Wenxing Pang

**Affiliations:** ^1^ College of Horticulture, Shenyang Agricultural University, Shenyang, China; ^2^ State Key Laboratory of Vegetable Biobreeding, Institute of Vegetables and Flowers, Chinese Academy of Agricultural Sciences, Beijing, China; ^3^ State Key Laboratory of Plant Cell and Chromosome Engineering, Institute of Genetics and Developmental Biology, Chinese Academy of Sciences, Beijing, China; ^4^ Institute of Vegetable Research, Liaoning Academy of Agricultural Sciences, Shenyang, China; ^5^ College of Plant Protection, Shenyang Agricultural University, Shenyang, China

**Keywords:** *Plasmodiophora brassicae*, Chinese cabbage, clubroot, transcription factor, intracellular

## Abstract

Clubroot disease, which is caused by the obligate biotrophic protist *Plasmodiophora brassicae*, leads to the formation of galls, commonly known as pathogen-induced tumors, on the roots of infected plants. The identification of crucial regulators of host tumor formation is essential to unravel the mechanisms underlying the proliferation and differentiation of *P. brassicae* within plant cells. To gain insight into this process, transcriptomic analysis was conducted to identify key genes associated with both primary and secondary infection of *P. brassicae* in Chinese cabbage. Our results demonstrate that the k-means clustering of subclass 1, which exhibited specific trends, was closely linked to the infection process of *P. brassicae*. Of the 1610 differentially expressed genes (DEGs) annotated in subclass 1, 782 were identified as transcription factors belonging to 49 transcription factor families, including bHLH, B3, NAC, MYB_related, WRKY, bZIP, C2H2, and ERF. In the primary infection, several genes, including the predicted *Brassica rapa* probable pectate lyase, RPM1-interacting protein 4-like, L-type lectin-domain-containing receptor kinase, G-type lectin S-receptor-like serine, *B. rapa* photosystem II 22 kDa protein, and MLP-like protein, showed significant upregulation. In the secondary infection stage, 45 of 50 overlapping DEGs were upregulated. These upregulated DEGs included the predicted *B. rapa* endoglucanase, long-chain acyl-CoA synthetase, WRKY transcription factor, NAC domain-containing protein, cell division control protein, auxin-induced protein, and protein variation in compound-triggered root growth response-like and xyloglucan glycosyltransferases. In both the primary and secondary infection stages, the DEGs were predicted to be *Brassica rapa* putative disease resistance proteins, L-type lectin domain-containing receptor kinases, ferredoxin-NADP reductases, 1-aminocyclopropane-1-carboxylate synthases, histone deacetylases, UDP-glycosyltransferases, putative glycerol-3-phosphate transporters, and chlorophyll a-binding proteins, which are closely associated with plant defense responses, biosynthetic processes, carbohydrate transport, and photosynthesis. This study revealed the pivotal role of transcription factors in the initiation of infection and establishment of intracellular parasitic relationships during the primary infection stage, as well as the proliferation and differentiation of the pathogen within the host cell during the secondary infection stage.

## Introduction

Clubroot disease, which is caused by the obligate biotrophic protist *Plasmodiophora brassicae*, affect the roots of cruciferous plants, leading to the formation of root galls that disrupt water and nutrient uptake (Voorrips et al., 2003). It is considered one of the most damaging diseases affecting crucifer crops worldwide, and can cause total yield loss under conducive conditions (Dixon, 2009a). The disease affects all cultivated Brassica species, including important vegetables and oilseed crops such as Chinese cabbage, cauliflower, cabbage, turnip, oilseed rape, and the model plant *Arabidopsis thaliana* (Dixon, 2009b).

A significant breakthrough in clubroot research was the unraveling of the pathogen life cycle and infection processes. Scientists have successfully identified the crucial stages in the development of this disease, including spore germination, primary infection, secondary infection, and gall formation. It is difficult to observe the clear time point between primary infection and secondary infection cause of the primary and secondary zoospores cannot be differentiated based on morphology (Kageyama and Asano, 2009). Feng et al. (2013a) reported that primary infections began to be noticed as early as 12 hours after inoculation (hai) with resting spores and secondary infections were observed at 72 hai. In recently, Liu et al. (2020) reported that the primary infection stage from 0 to 7 days post inoculation (DPI)and the secondary infection stage from 7 to the resting spore formation. Starch accumulation in infected hosts provides *P. brassicae* with carbon and energy during infection (Ikegami et al., 1984; Schuller et al., 2016; Ma et al., 2022). This understanding has paved the way for targeted interventions to disrupt these processes and reduce the disease severity.

Genetic studies have also played a crucial role in clubroot research, as researchers have identified and characterized multiple resistance genes in cruciferous crops that confer resistance to specific pathotypes of *P. brassicae* (Pang et al., 2020). Advancements in molecular techniques have facilitated the identification and characterization of genes involved in clubroot susceptibility and resistance (Ueno et al., 2012; Hatakeyama et al., 2013; Pang et al., 2022; Wang et al., 2023). Effective clubroot management relies heavily on the use of resistant cultivars because chemical and cultural controls have limited effectiveness against this soil-borne disease. However, the resistance conferred by major clubroot resistance (CR) genes is often quickly overcome by the prevalence of pathogenic strains of *P. brassicae* as a result of selection pressure (Kuginuki et al., 1999; Strelkov et al., 2016; Struck et al., 2022). Therefore, it is crucial to make efforts to understand the molecular mechanisms underlying clubroot pathogenesis and CR in order to develop durable clubroot resistance and improve management strategies.

Over the past decade, transcriptomic studies utilizing microarrays and RNA sequencing have generated extensive datasets that offer novel insights into the molecular basis of clubroot infection and defense responses in cruciferous hosts (Ciaghi et al., 2019; Zhou et al., 2020). Extensive transcriptomic reprogramming occurred during the secondary phase of clubroot development and coincided with gall formation. Galls develop abnormally enlarged cells with thin cell walls, large vacuoles, dense cytoplasmic bodies, and highly proliferating mitochondria and plastids (Ludwig-Müller et al., 2009; Dodueva et al., 2020). Feng et al. (2013b) reported that the differentially expressed gene expression patterns in primary and secondary zoospores investigated by dot-blot and qPCR. Microarray analysis of Arabidopsis has identified over 3,900 differentially expressed genes (>2-fold change) in infected gall tissues, whereas RNA-seq has identified over 4,500 differentially expressed transcripts (Agarwal et al., 2011; Jubault et al., 2013). The upregulated pathways in galls included JA, ET, auxin, CK, and brassinosteroid (BR) hormone signaling, which stimulate cell enlargement and division. Genes involved in cell wall modification, cytochrome P450s, transporters, and DNA replication/repair were also upregulated. Conversely, the genes associated with photosynthesis, sulfur/glucosinolate metabolism, defense responses, and cell death were strongly downregulated, indicating extensive metabolic reprogramming. Comparative analysis of Arabidopsis transcriptomes at 10, 14, 21, and 28 DPI revealed dynamic temporal changes, with the most significant alteration occurring at 21 DPI, coinciding with extensive gall formation (Agarwal et al., 2011). In late-stage infection (35 DPI), Shi et al. (2014) identified 515 *Arabidopsis* genes that were differentially expressed and shared between *B. napus* and *B. rapa*. These included genes involved in cell wall modification, hormone signaling, and secondary metabolism. Phenylpropanoid biosynthesis is exclusively induced in Brassica species, whereas photosynthetic genes are repressed solely in Arabidopsis. These analyses revealed the common induction of certain cellular processes, such as cell wall remodeling and hormone signaling, but also indicated divergence in specific responsive genes during clubroot infection across crucifer hosts. Additionally, they demonstrated a stronger correlation between transcriptional changes during the latter stages of disease progression than during the early stages.

Comparative transcriptomics of clubroot-resistant and clubroot-susceptible genotypes have facilitated the identification of candidate resistance genes and pathways. Chu et al. (2014) observed a higher upregulation of genes associated with ET signaling, glutathione-S-transferases, and trehalose biosynthesis in resistant *B. rapa* lines than in susceptible lines at 10 DPI. Hosts of crucifers and *P. brassicae* interactions in clubroot has been reviewed in which clubroot pathogenesis and host resistance were well discussed (Feng et al., 2014). Furthermore, RNA-seq analysis of two *B. napus* lines with varying resistance revealed 618 differentially expressed genes at 21 DPI, which were related to cell organization, biotic stress response, hormone signaling, and glucosinolate biosynthesis (Zhang et al., 2016). The more resistant line exhibited increased expression of the camalexin biosynthesis genes PAD3 and CYP71A13, which corresponded to higher levels of camalexin.

These findings contribute to a better understanding of the mechanisms involved in plant defense responses and susceptibility to clubroot. Over the past ten years, research on clubroot disease has made noteworthy progress in various areas, such as pathogen biology, genetics, and management strategies (Zhang et al., 2023). These advancements have not only improved our comprehension of the disease, but have also provided valuable tools and techniques for effective disease control. However, continuous research efforts are required to advance our understanding of pathogen evolution and adaptation, thereby ensuring sustainable management options for cruciferous crop production.

## Materials and methods

### Plant materials and *P. brassica* inoculation

In this study, a highly susceptible Chinese cabbage inbred line ‘325’ was used. The ‘325’ line was inoculated with P. brassicae isolate ‘SCDY-57,’ identified as pathotype Pb1 according to the Sinitic clubroot differential set (Pang et al., 2020). The P. brassicae resting spore preparation and inoculation processes followed the methodology described by Pang et al. (2020). Briefly, the galls were ground in sterile distilled water using a homogenizer, and the resulting mixtures were filtered through eight layers of cheesecloth. Resting spores were collected by centrifugation at 2,500g and quantified using a hemocytometer (Neubauer improved, Marienfeld, Germany). The concentration of the resting spores was adjusted to 1 × 10^7^/ml, and 1 ml of the suspension was inoculated into 10-day-old seedlings of each plant. Plants treated with 1 ml distilled water were used as mock controls.

### Transcriptome sample preparation, total RNA isolation, and transcriptome sequencing

Tissue sampling was performed at 3, 7, 14, 21, 28, and 35 DPI with P. brassicae isolate ‘SCDY-57’ or distilled water according to Ma et al. (2022). Roots of 20 individual plants were sampled at each time point and used for RNA isolation. Total RNA was extracted from infected and mock control root samples using TRIzol™ reagent (Invitrogen, Carlsbad, USA). Additional RNA quality was determined using a NanoDrop spectrophotometer (Thermo Scientific, Waltham, MA, USA). Sequencing libraries were generated using the NEBNext UltraTM RNA Library Prep Kit for Illumina [New England Biolabs (NEB), Ipswich, MA, USA], following the manufacturer’s recommendations, and index codes were added to attribute sequences for each sample. Sequencing was performed on an Illumina HiSeq 2500 platform (Illumina, San Diego, CA, USA) by Annoroad Gene Technology Co., Ltd. (Beijing, China).

### Preprocessing and *de novo* assembly

Raw data were cleaned by removing adapter sequences, N-sequences, and low-quality reads. Reference genomes of Brassica rapa (Brapa_genome_v3.0) and P. brassicae downloaded from the Brassicaceae Database and NCBI were used in this study (Stjelja et al., 2019; Chen et al., 2022). Bowtie2 v2.2.3 was used to build the genome index, and Clean Data were aligned to the reference genome using HISAT2 v2.1.0. The read Count for each gene in each sample was determined using HTSeq v0.6.0, and fragments per kilobase million mapped reads (FPKM) were calculated to estimate the expression level of genes in each sample. DEGseq was used for differential gene expression analysis. Genes with q ≤ 0.05 and |log2_ratio|≥1 are identified as differentially expressed genes (DEGs). The TBtools software (Chen et al., 2020) was used for the Venn map and heat map analysis according to the RNA-Seq data in this study.

### Unigene annotation and classification

Functional and pathway enrichment of proteins encoded by the candidate genes was analyzed. Gene Ontology (GO, http://geneontology.org/) enrichment of DEGs was implemented using the hypergeometric test, in which the p-value was calculated and adjusted as a q-value, and the data background was genes in the whole genome. GO terms with q<0.05 were considered to be significantly enriched. GO enrichment analysis revealed the biological functions of DEGs. The KEGG enrichment of DEGs was implemented using the hypergeometric test, in which the p-value was adjusted by multiple comparisons as q-values. KEGG terms with q<0.05 were considered to be significantly enriched.

### Quantification of gene expression levels and differential expression analysis

PrimeScript™ RT Reagent Kit (TaKaRa, Beijing, China) was used for cDNA synthesis according to the manufacturer’s instructions. The Primer 3.0 online program was used to design the primers for qRT–PCR; the primer information is listed in **Supplementary Table 5**. The qRT–PCR was conducted in TB Green^®^ Premix Ex Taq™ II FAST qPCR (TaKaRa, Beijing, China) with the CFX96™ Real-Time System. All experiments were performed in triplicate. The relative expression levels of the genes were determined by the 2^−ΔΔCT^ method (Livak and Schmittgen, 2001), and the sample from each time point for distilled water treatment was used as the control.

### Data analysis

Data analyses were performed using the SPSS statistical package (SPSS, Chicago, IL, USA). Analysis of variance (ANOVA) was conducted to evaluate the treatments. If a significant treatment effect was identified (P = 0.05), Duncan’s Multiple Range Test was used to determine significant differences at P < 0.05.

## Results

### Transcriptome analyses

A total of 12 root samples were collected from Chinese cabbage inbred line ‘325’ at 3, 7, 14, 21, 28, and 35 days post-treatment with P. brassicae isolate ‘SCDY-57’ as well as distilled water. The samples were named D3Pb, W1Pb, W2Pb, W3Pb, W4Pb, and W5Pb, and D3CK, W1CK, W2CK, W3CK, W4CK, and W5CK for 3, 7, 14, 21, 28, and 35 days after treated with P. brassicae and distilled water, respectively. Twelve libraries were constructed and analyzed, resulting in clean Q30 base-rate values ranging from 93.77%–94.31%. Overall, 516 million good-quality reads were obtained, with mapping rates to the B. rapa genome ranging from 35.61% to 90.51% (**Table 1**). The mapping rate greatly increased from 0.0078% to 58.37% in P. brassicae genome from 3 to 35 days after P. brassicae inoculation. In total, 30495 and 10518 genes were predicted from W5Pb for B. rapa and P. brassicae, respectively.

**Table 1:** The clean reads mapping rate and annotated gene number of the RNA-Seq data were compared to the reference genome of *Brassica rapa* and *Plasmodiophora brassicae*.

NO.	Sample name	Total clean reads	*Brassica rapa*		*Plasmodiophora brassicae*	
			Mapping rate^A^	Total gene number	Mapping rate^B^	Total gene number
1	D3CK	40,145,846	0.8936	31001	–	–
2	D3Pb	44,413,690	0.9004	32156	7.77E-05	180
3	W1CK	45,020,494	0.9051	32164	–	–
4	W1Pb	40,464,846	0.9031	31981	0.0004	2238
5	W2CK	40,308,672	0.8781	31730	–	–
6	W2Pb	42,711,550	0.8900	32198	0.0197	8071
7	W3CK	44,778,000	0.8986	32277	–	–
8	W3Pb	43,520,202	0.7752	31959	0.1387	9280
9	W4CK	44,358,100	0.8723	31202	–	–
10	W4Pb	43,316,456	0.5481	31127	0.3601	10416
11	W5CK	41,024,024	0.8988	31511	–	–
12	W5Pb	45,517,750	0.3561	30495	0.5837	10518

D3Pb, W1Pb, W2Pb, W3Pb, W4Pb, W5Pb, and D3CK, W1CK, W2CK, W3CK, W4CK, and W5CK indicate *P. brassicae* and distilled water treatments at 3, 7, 14, 21, 28, and 35 days, respectively. ^A^Mapping rate indicated the total clean reads map to the *Brassica rapa* (Brapa_genome_v3.0) genome; ^B^Mapping rate indicated the total clean reads map to the Plasmodiophora brassicae genome. -, indicated that no data generated.

**Figure 1:**
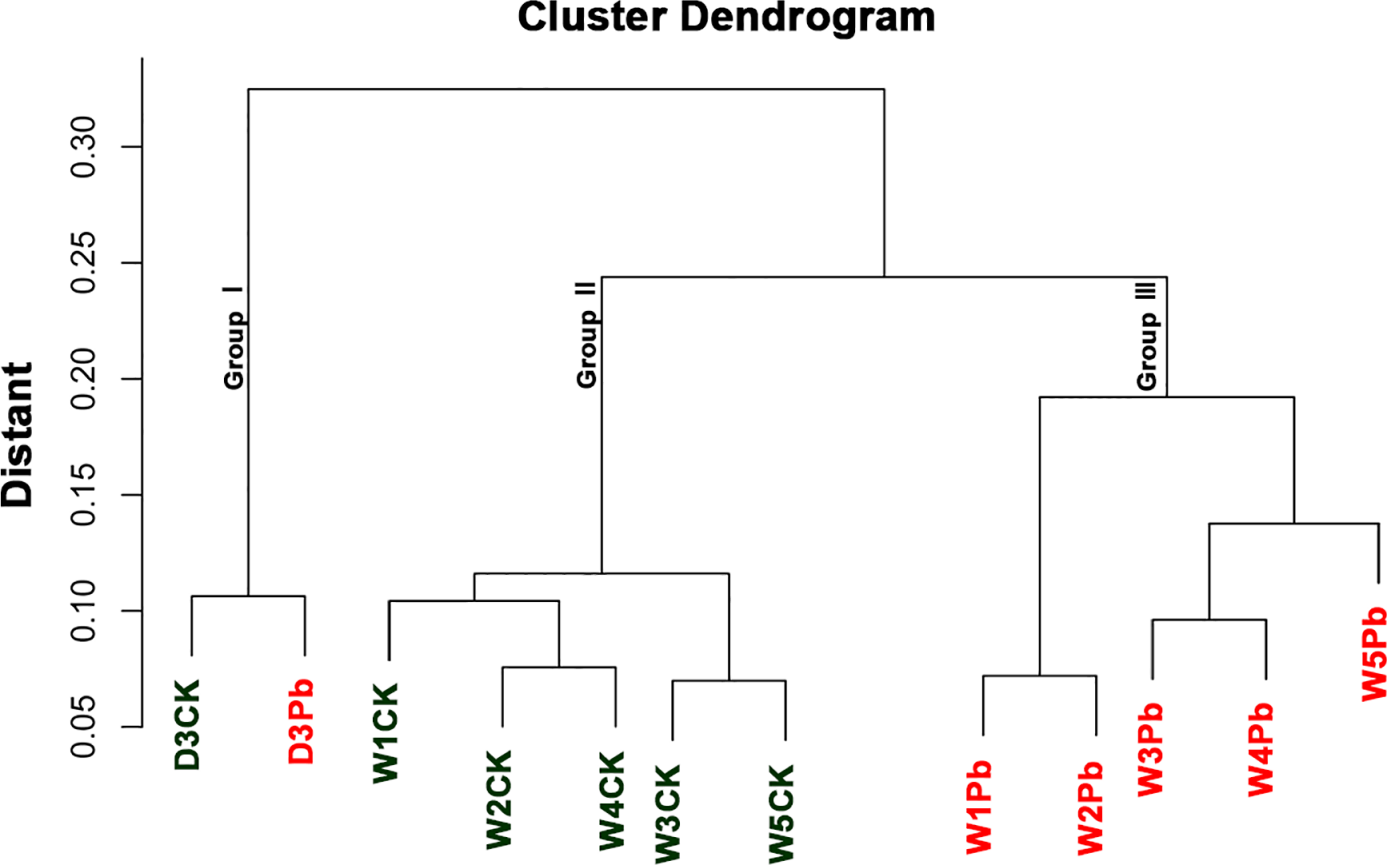
Cluster dendrogram and grouping information of 12 samples.

The sample cluster revealed that 12 samples were grouped into three distinct clusters (**Figure 1**). Samples D3CK and D3Pb formed a separate cluster that was distinct from that of the other samples. Furthermore, the remaining 10 samples were divided into two groups, with W1Pb, W2Pb, W3Pb, W4Pb, and W5Pb representing the samples treated with P. brassicae, and W1CK, W2CK, W3CK, W4CK, and W5CK representing the samples treated with distilled water.

### Differently expressed genes (DEGs)

**Figure 2:**
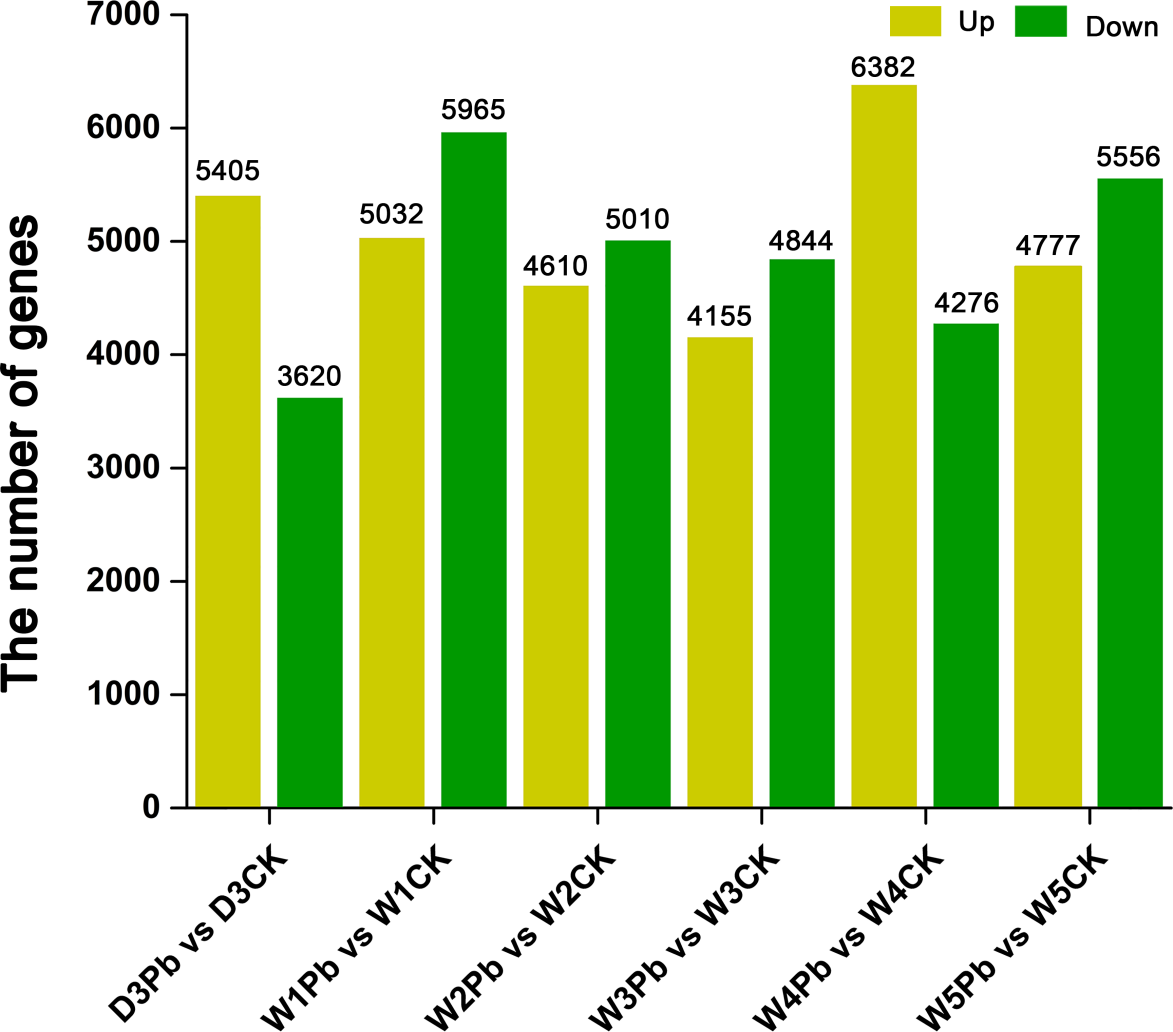
Numbers of differentially expressed genes (DEGs) obtained from *P. brassicae* inoculation compared to distilled water at 3, 7, 14, 21, 28, and 35 days after treatment.

A total of 9025, 10997, 9620, 8999, 10658, and 10333 DEGs were identified in response to P. brassicae inoculation compared to distilled water at 3, 7, 14, 21, 28, and 35 days post-treatment (**Figure 2**). The downregulated genes were upregulated more in all P. brassicae treatments than in distilled water, except at 3 and 28 d after treatment.

**Figure 3:**
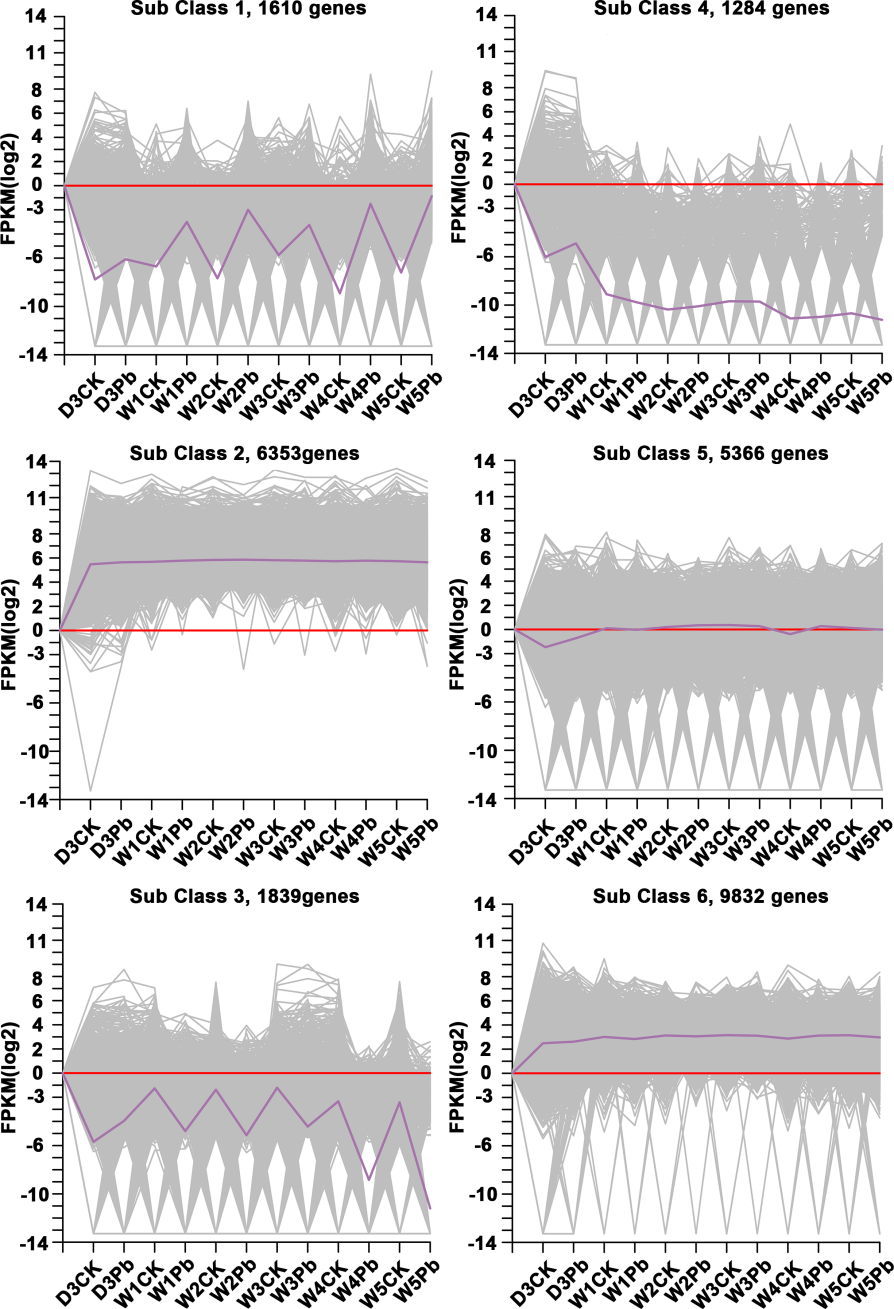
Clusters obtained by k-means cluster analysis.

**Figure 4:**
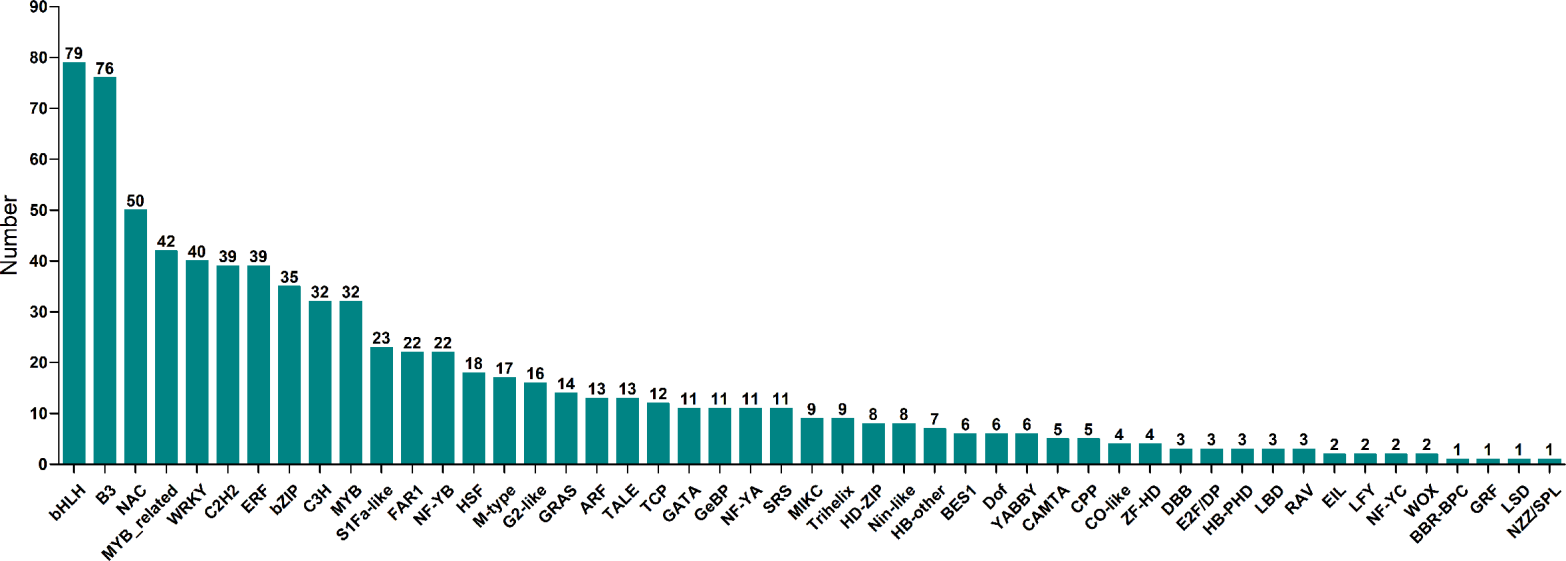
Transcription factors number of 49 transcription factor family annotated in sub class 1.

K-means clustering analysis of the DEGs revealed that these genes were related to processes associated with the infection of P. brassicae (**Figure 3**). The results showed that the k-means clustering of sub-classes 1 and 3 with certain trends were associated with the infection of P. brassicae. However, sub-class 1 had a much closer relationship with the process of P. brassicae infection than sub-class 3. Of the 1610 DEGs annotated in subclass 1, 782 were characterized as transcription factors. These 782 genes belonged to 49 transcription factor families, including basic helix-loop-helix (bHLH), B3, NAC (no apical meristem (NAC), MYB_related, WRKY, bZIP (basic region/leucine zipper motif (bZIP), C2H2, and ERF(Ethylene response factors) etc. (**Figure 4**). Most genes were annotated to the transcription factor family bHLH, and 79 genes were detected.

### Functional annotation of sub class 1

**Figure 5:**
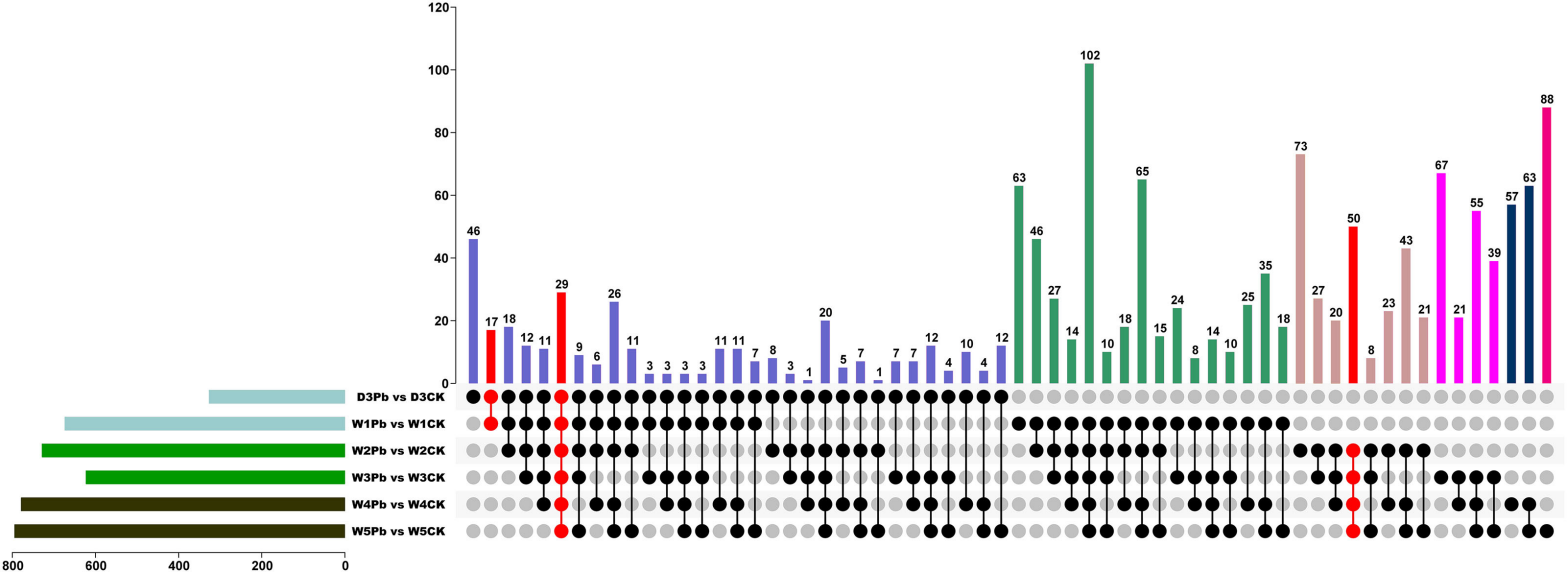
Venn diagram of differentially expressed genes (DEGs) from *P. brassicae* inoculation compared to distilled water at 3, 7, 14, 21, 28, and 35 days after treatment.

To explore the functional genes involved in P. brassicae infection, DEGs in subclass 1 were chosen for further analysis. **Figure 5** shows the Venn map of the DEGs obtained from subclass 1. There were 29 overlapping genes at all-time points. Moreover, 17 and 50 genes overlapped at the primary (3 and 7 DAI, respectively) and secondary (14, 21, 28, and 35 DAI) infection stages (**Figure 5**).

**Figure 6:**
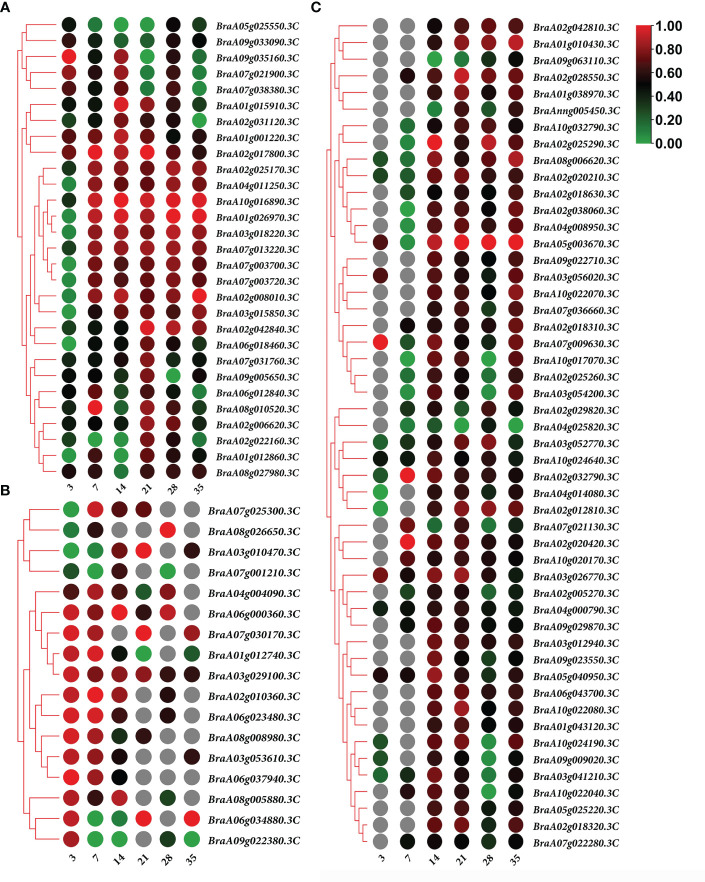
Heatmap of gene expression. **(A)** 29 overlapping differentially expressed genes (DEGs) expression heatmap in both of the primary and secondary infection stages. **(B)** 17 overlapping DEGs expression heatmap in primary infection stage. **(C)** 50 overlapping DEGs expression heatmap in the secondary infection stage. Notes: Numbers 3, 7, 14, 21, 28, and 35 indicate the different stages at 3, 7, 14, 21, 28, and 35 days after treatment, respectively.

In both the primary and secondary infection stages, the 29 overlapping DEGs were predicted to be Brassica rapa putative disease resistance proteins, L-type lectin-domain-containing receptor kinases, ferredoxin–NADP reductases, 1-aminocyclopropane-1-carboxylate synthases, histone deacetylases, UDP-glycosyltransferases, putative glycerol-3-phosphate transporters, and chlorophyll a-b binding proteins, which are closely related to plant defense responses, biosynthetic processes, carbohydrate transport, and photosynthesis (**Figure 6A**; **Supplementary Table 1**).

The predicted Brassica rapa probable pectate lyase, RPM1-interacting protein 4-like, L-type lectin-domain-containing receptor kinase, Brassica napus G-type lectin S-receptor-like serine, Brassica rapa photosystem II 22 kDa protein and MLP-like protein were all upregulated during primary infection (**Figure 6B**; **Supplementary Table 2**). Meanwhile, the predicted Brassica rapa cation/H(+) antiporter and Brassica napus uncharacterized LOC106429977 were downregulated during primary infection (**Figure 6B**; **Supplementary Table 2**).

In the secondary infection stage, 45 out of 50 overlapping DEGs were upregulated, including predicted as Brassica rapa endoglucanase, long-chain acyl-CoA synthetase, probable WRKY transcription factor, NAC domain-containing protein, cell division control protein, auxin-induced protein, protein variation in compound triggered root growth response-like, and Brassica napus xyloglucan glycosyltransferase (**Figure 6C**; **Supplementary Table 3**). Only 5 DEGs BraA02g029820.3C, BraA07g021130.3C, BraA09g063110.3C, BraA04g025820.3C and BraAnng005450.3C, identified as Brassica rapa kinesin-like protein NACK1, cell division control protein, and uncharacterized, were downregulated at certain time points (**Figure 6C**; **Supplementary Table 3**).

### KEGG analysis of identified DEGs

**Figure 7:**
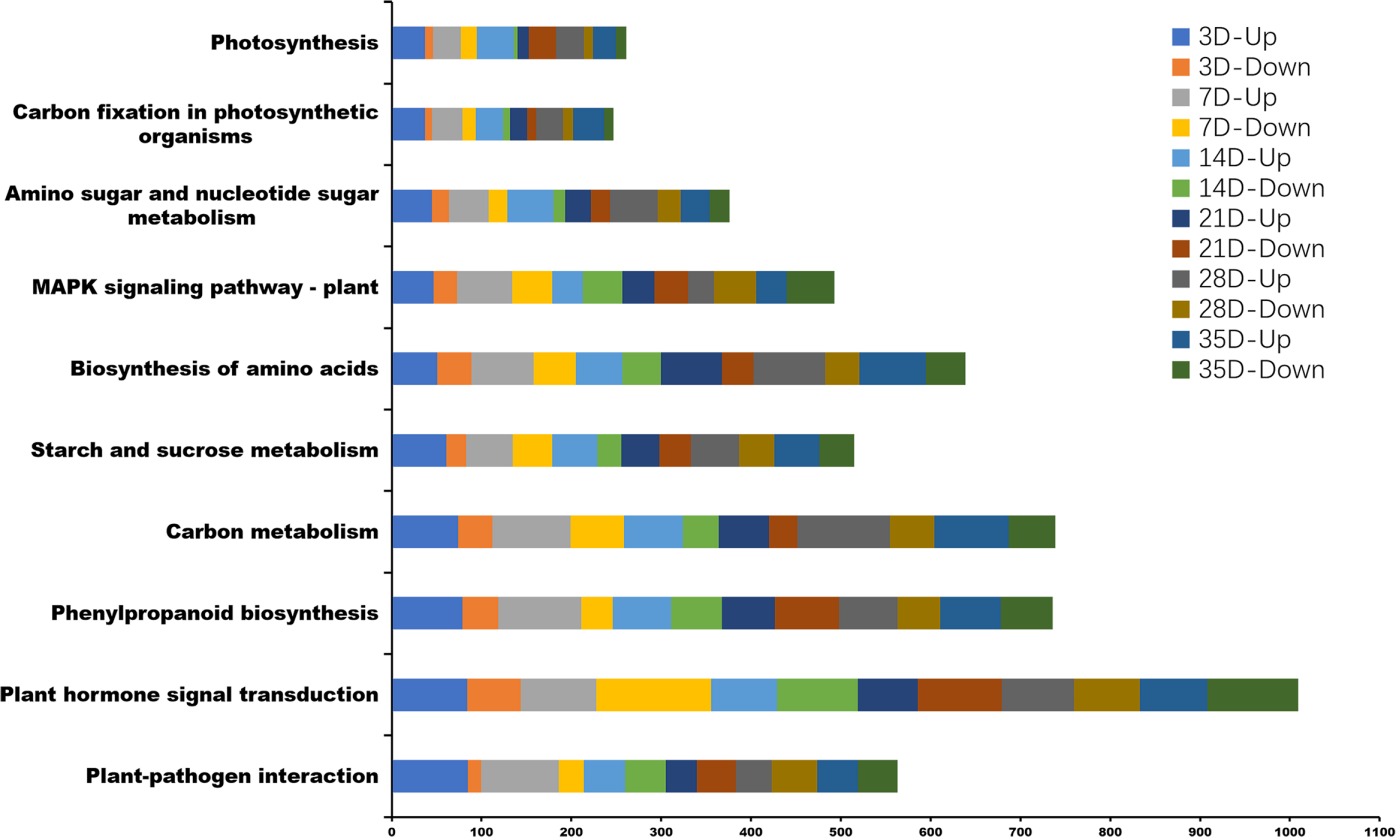
The top 10 Kyoto Encyclopedia of Genes and Genomes (KEGG) pathway enrichment analysis between *P. brassicae* inoculation compared to distilled water at 3, 7, 14, 21, 28, and 35 days after treatment.

Kyoto Encyclopedia of Genes and Genomes (KEGG) pathway enrichment analysis, 131, 131, 132, 131, 132, and 133 KEGG pathways were associated with the infection of P. brassicae at 3, 7, 14, 21, 28, and 35 days after treatment. The top 10 enriched pathways are shown in **Figure 7**; **Supplementary Table 4**. KEGG analysis revealed significant enrichment in plant hormone signal transduction, plant-pathogen interactions, starch and sucrose metabolism, MAPK signaling, cysteine and methionine metabolism, carbon metabolism, and phenylpropanoid biosynthesis pathways, all of which are related to the infection process of P. brassicae.

### Verification of DEGs involved in different *P. brassicae* infection stages

**Figure 8:**
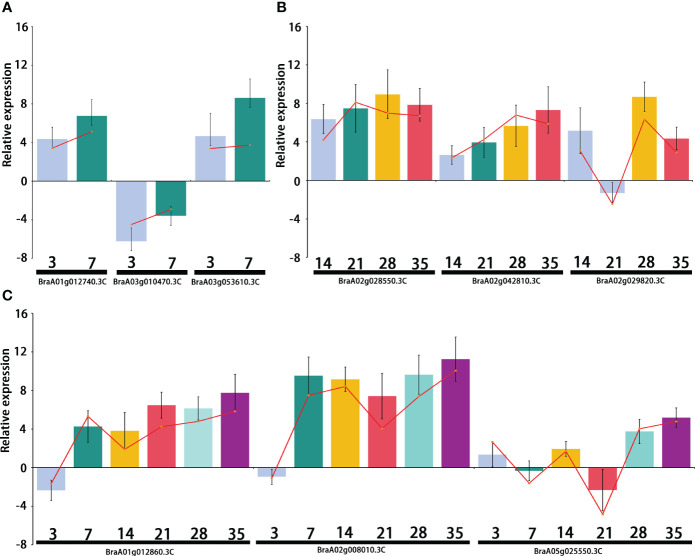
Relative expression of the differentially expressed genes (DEGs) selected from the primary infection, secondary infection, and primary and secondary infection stages, respectively. **(A)** Relative expression of DEGs from the primary infection stage; **(B)** Relative expression of DEGs from the secondary infection stage; **(C)** Relative expression of DEGs from the primary and secondary infection stages. Bar chat graph represents the gene expression by comparing *P. brassicae* inoculation to distilled water treatment from qRT-PCR at different stages, and the red line represents the gene expression from RNA-seq.

Differentially expressed transcription factors were selected from primary, secondary, and primary and secondary infection stages. Three genes were selected at each stage for quantitative real-time PCR analysis. The primers used for qRT-PCR are listed in **Supplementary Table 5**. The qRT-PCR results showed the same expression pattern as the RNA-Seq data (**Figure 8**). During the primary infection stage, *BraA01g012740.3C* (*Brassica rapa* probable pectate lyase 16) and *BraA03g053610.3C* (*Brassica napus* G-type lectin S-receptor-like serine/threonine-protein kinase) were significantly upregulated at 3 and 7 DPI. However, *BraA03g010470.3C* (*Brassica rapa* cation/H(+) antiporter 9-like) was significantly downregulated at 3 and 7 DPI (**Figure 8A**). During the secondary infection stage, the WRKY transcription factor BraA02g028550.3C (*Brassica rapa* long-chain acyl-CoA synthetase 5-like) and *BraA02g042810.3C* (Brassica rapa histone deacetylase 5-like) were significantly upregulated (**Figure 8B**). Both *BraA01g012860.3C* (*Brassica rapa* putative cysteine-rich receptor-like protein kinase) and *BraA02g008010.3C* (*Brassica rapa* putative disease resistance protein) were downregulated at 3DPI and then, upregulated, 7,14,21,28 and 35 DPI (**Figure 8C**).

## Discussion

Infection with P. brassicae leads to increased cell division in both the cambium and the phloem parenchyma of Arabidopsis (Kobelt et al., 2000). Tumors in higher plants are abnormal tissue outgrowths resulting from the uncontrolled proliferation of a group of cells (Dodueva et al., 2020). Therefore, clubs that form on the roots of infected plants caused by the obligate biotrophic protist P. brassicae also called tumors, were described by Dodueva et al. (2020) in a review. Clubroot causes considerable economic damage owing to reduced crop yields in cultivated cruciferous crops. The prevention and management of clubroots has become a global challenge. Transcriptomic and genomic studies of the interactions between P. brassicae and cruciferous plants have provided valuable insights into the molecular mechanisms underlying these interactions (Zhao et al., 2017; Irani et al., 2018; Li et al., 2023). Studies have identified differentially expressed genes and signaling pathways associated with the response of cruciferous plants to P. brassicae infection (Grsic-Rausch et al., 2000; Devos et al., 2005; Ma et al., 2022).

The cell wall provides the first line of defense in plants and plays an important role in disease resistance (Wan et al., 2021). Microorganisms have evolved various strategies to break down cell walls. Pectin is a major component of primary cell walls and plays an important role in cell wall formation in higher plants. During the primary infection, the predicted *B. rapa* probable pectate lyase gene *BraA01g012740.3C* was upregulated by infection with *P. brassicae* (**Figure 6B**). *BraA01g012740.3C* encodes an HD-ZIP family protein that activates Poly(1,4-alpha-D-galacturonate)(n) via the unsaturated D-galacturonate pathway. A series of pectin enzymes are secreted by microorganisms to directly break down de-esterified HG, and infection attempts have been reported (Lionetti et al., 2012). Moreover, Pst DC3000 hijacks the host signaling pathway, which induces cell wall remodeling during plant development in *Arabidopsis* (Wang et al., 2017). IDL6 is upregulated upon infection with Pst DC3000, which then activates the HAE/HSL2 pathway, thereby promoting pectin degradation. These results indicated that Pst DC3000 can enhance infection by hijacking the IDL6-HAE/HSL2-ADPG2 signaling pathway (Wang et al., 2017). RPM1-interacting protein 4 (RIN4) is a conserved plant immunity regulator that can be modified by pathogenic effector proteins that plays an important role in both PAMP-triggered (PTI) and effector-triggered immunity (ETI) (Zhao et al., 2021). Kim et al. (2005) showed that RIN4 is a negative regulator of regulator of PAMP signaling, as the overexpression of RIN4 results in reduced defense responses. *BraA02g010360.3C*, predicted to be *B. rapa* RPM1-interacting protein 4-like, was highly upregulated by infection with *P. brassicae* during primary infection (**Figure 6B**). Moreover, L-type lectin domain-containing receptor kinase (*BraA03g029100.3C*, S1Fa-like), *B. napus* G-type lectin S-receptor-like serine (*BraA03g053610.3C*,TALE), and *B. rapa* photosystem II 22 kDa protein (*BraA08g005880.3C*) were upregulated at the primary infection stage. L-type lectin receptor kinases (LecRKs) recognize a variety of invasion patterns because of their large diversity. LecRKs are key players in plant immunity; however, their functions in plant defense are not well understood (Wang and Bouwmeester, 2017). The G-type lectin S-receptor-like serine/threonine protein kinase has been identified as a positive regulator of salt stress (Sun et al., 2013). Major latex-like proteins (MLP) confer resistance to pathogens by inducing pathogenesis-related protein genes. The MLP-like protein (*BraA08g026650.3C*) was downregulated at 3 DPI in *P. brassicae*. All of these DEGs were closely related to pathogen invasion induced by the infection of *P. brassicae*, offering insights for future investigations.

*P. brassicae* can manipulate Brassicaceae hosts by hijacking plant carbohydrate metabolism pathways to generate a strong physiological sink, such as accumulating abundant starch grains in infected roots (Malinowski et al., 2012; Ma et al., 2022). Our study showed that putative glycerol-3-phosphate transporter (*BraA08g010520.3C*), *B. rapa* endoglucanase 9-like (*BraA02g020420.3C*, WRKY), and long-chain acyl-CoA synthetase *(BraA02g028550.3C*, WRKY) were upregulated during *P. brassicae* secondary infection stage. A recent study reported that glucose transporters and glucose content significantly increase during the late stages of root infection (Kong et al., 2022). Moreover, previous studies have shown that the upregulation of the MEX1 maltose transporter and starch synthesis pathway (BrAGPS2 and BrISA2b) is activated by the growing *P. brassicae* plasmodia to mediate the energy supply from the host to the pathogen (Badstöber et al., 2020; Ma et al., 2022).

In the present study, transcription factors related to carbohydrate synthesis and transportation were found to play important roles in the invasion and proliferation of *P. brassicae.* Transcription factors such as *BraA02g018320.3C*, *BraA03g056020.3C*, *BraA07g022280.3C*, *BraA10g022040.3C*, *BraA10g022070.3C*, *BraA10g022080.3C*, *BraA10g024190.3C* and *BraA10g024640.3C* all belong to the bHLH family. These genes annotated as *B. rapa* protein variation in compound-triggered root growth response-like, *B. rapa* lectin domain-containing receptor kinase VI.3-like, *B. rapa* auxin-induced protein 15A-like, *B. rapa* transcription factor PRE2, *B. rapa* cytochrome P450 were upregulated during the secondary infection stage. Lectins serve as sugar code readers and reversibly bind to specific carbohydrates (Naithani et al., 2021). Evidence has shown that L-type lectin receptor kinase is involved in the resistance response to *the pathogenic oomycetes P. infestans and P. capsici and fungus A. brassicicola (* Wang et al., 2014). Auxins are also involved in invasion and gall formation during *P. brassicae* infection (Malinowski et al., 2016), and the auxin-induced protein-encoding gene 15A-like (*BraA10g022040.3C*, *BraA10g022070.3C*, *BraA10g022080.3C*) was upregulated, strengthening the result that auxin and cytokines play a key role during gall formation (Devos et al., 2006). The cytochrome P450 (CYP) superfamily catalyzes a wide range of reactions and plays important roles in several fundamental biological processes, such as steroid synthesis, fatty acid metabolism, and chemical defense (Pankov et al., 2021). A total of 258 non-redundant P450 genes have been identified, and P450 genes may play essential roles in pathogen-triggered immunity (PTI) in Chinese cabbage (Zhang et al., 2021).

In the context of *Plasmodiophora brassicae* infection, transcription factor families including bHLH, B3, NAC, MYB_related, WRKY, bZIP, C2H2, and ERF regulate the expression of genes related to defense responses, cell wall modifications, hormone signaling, and other processes involved in the plant’s defense mechanisms against the pathogen. Their coordination is essential for orchestrating an effective response to the infection and is likely crucial for the plant’s ability to combat the pathogen. Overall, transcript and genomic studies have provided valuable insights into the molecular mechanisms underlying interactions between P. brassicae and cruciferous plants. Understanding these molecular mechanisms can help to develop strategies for the management and control of clubroot diseases. In summary, this interaction involves complex changes in gene expression in both organisms, with the pathogen actively modulating plant defense and development through the secretion of effector proteins. Further research on the transcription factors related to carbohydrate metabolism pathways will provide more evidence for P. brassicae host manipulation in cruciferous crops.

## Data availability statement

The datasets generated and analyzed during this study are available on reasonable requests from the corresponding authors. The RNA-Seq data of this study has been deposited at NCBI under the BioProject ID PRJNA1060352.

## Author contributions

SM: Data curation, Formal Analysis, Writing – original draft. XY: Data curation, Formal Analysis, Writing – original draft. YP: Data curation, Writing – review & editing. SL: Data curation, Writing – review & editing. XW: Data curation, Writing – review & editing. JJ: Data curation, Writing – review & editing. YL: Data curation, Writing – review & editing. WP: Data curation, Funding acquisition, Project administration, Resources, Writing – review & editing.
